# Supplementary material for: Human cortical encoding of pitch in tonal and non-tonal languages
Source: Nat Commun. 2021 Feb 19;12:1161. doi: 10.1038/s41467-021-21430-x (PMC7896081; doi:10.1038/s41467-021-21430-x)
Supplement: Supplementary file 1 — Supplementary Information [file 41467_2021_21430_MOESM1_ESM.pdf]

Supplementary Information

Human cortical encoding of pitch in tonal and non-tonal languages

Li *et al.*

Supplementary Figures

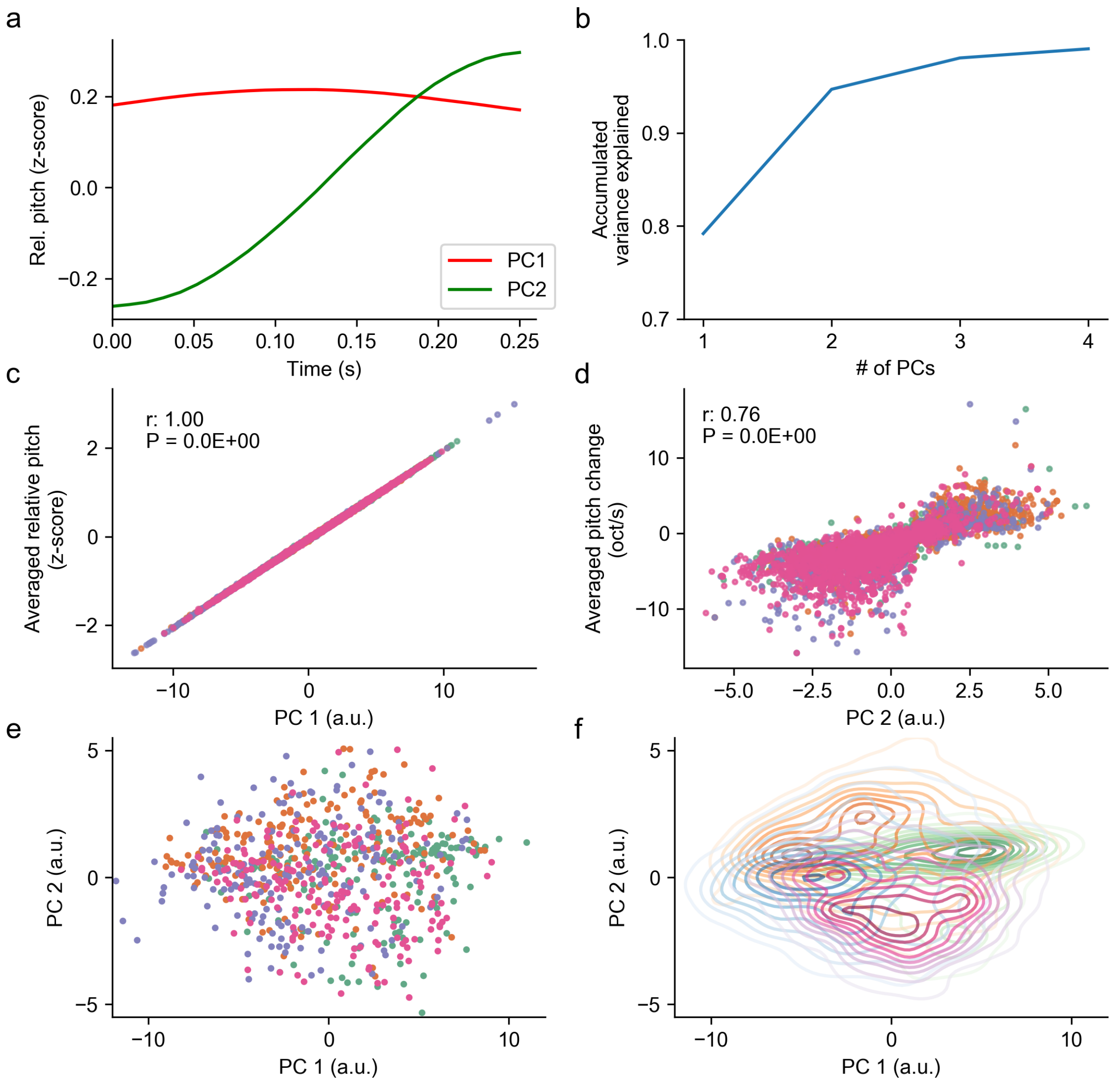

Supplementary Figure 1. Principal component analysis (PCA) on the pitch contours in Mandarin speech. (a) The time course of the first 2 PCs of the relative pitch contours from all 4 lexical tones; **(b)** the accumulated proportion of variance in the pitch contour explained by the first n PCs. **(c)** The correlation between the first PC and the average relative pitch height for tone exemplars. Each dot is a single tone exemplar, x-axis corresponds to the PC score (loading) along the first PC dimension, and y-axis corresponds to the average relative pitch height of the exemplar across time. **(d)** The correlation between the second PC and the average pitch change for tone exemplars. Each dot is a single tone exemplar, x-axis corresponds to the PC score (loading) along the second PC dimension, and y-axis corresponds to the average pitch change of the exemplar across time. **(e)** The scatter plot of pitch contours of the 4 lexical tones (200 exemplars from each tone) in the space of the first 2 PCs; **(f)** the corresponding probability density of the distributions of the 4 lexical tones in the PC space.

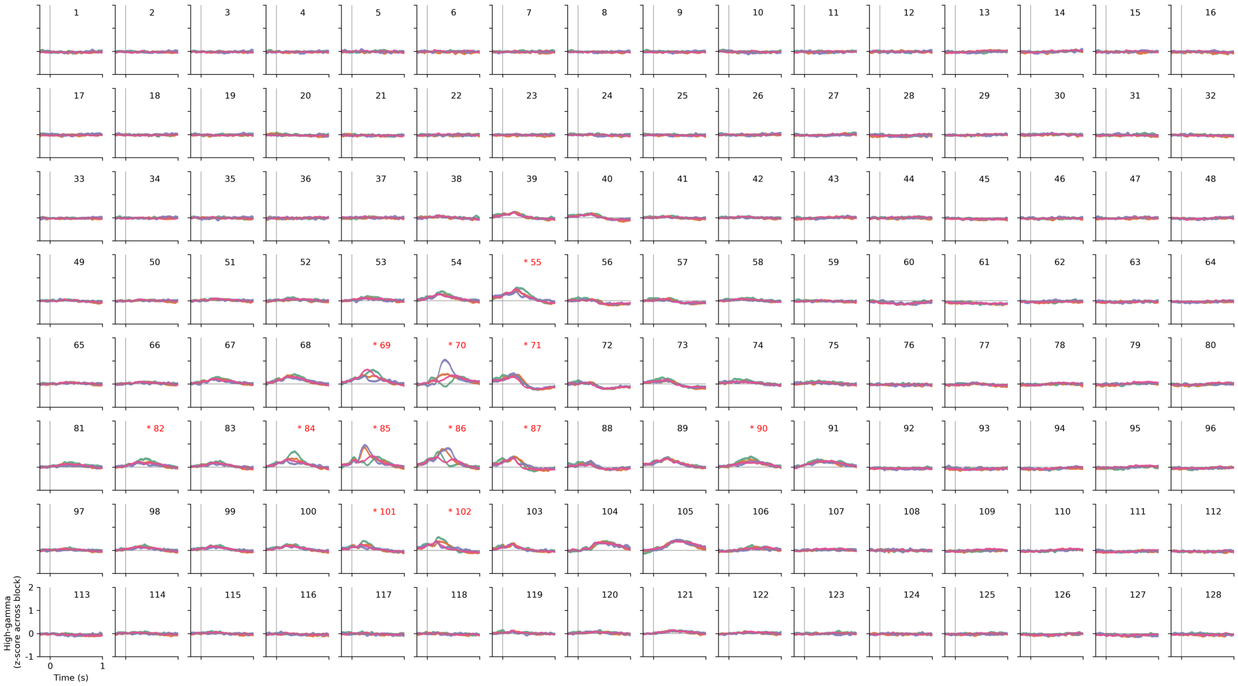

Supplementary Figure 2. The averaged event-related high-gamma activity for Mandarin lexical tones in single electrodes from an example participant (Subject M2). Each panel corresponds to a single electrode. Average neural activity (z-scored across entire recording block; shaded area represents mean ± s.e.m.) in response to each tone was plotted, aligned to vowel onset. Red * indicates electrodes with significantly different patterns between lexical tones (*P* < 0.05, F-test, two-sided, Bonferroni corrected for multiple comparisons across all time points and electrodes).

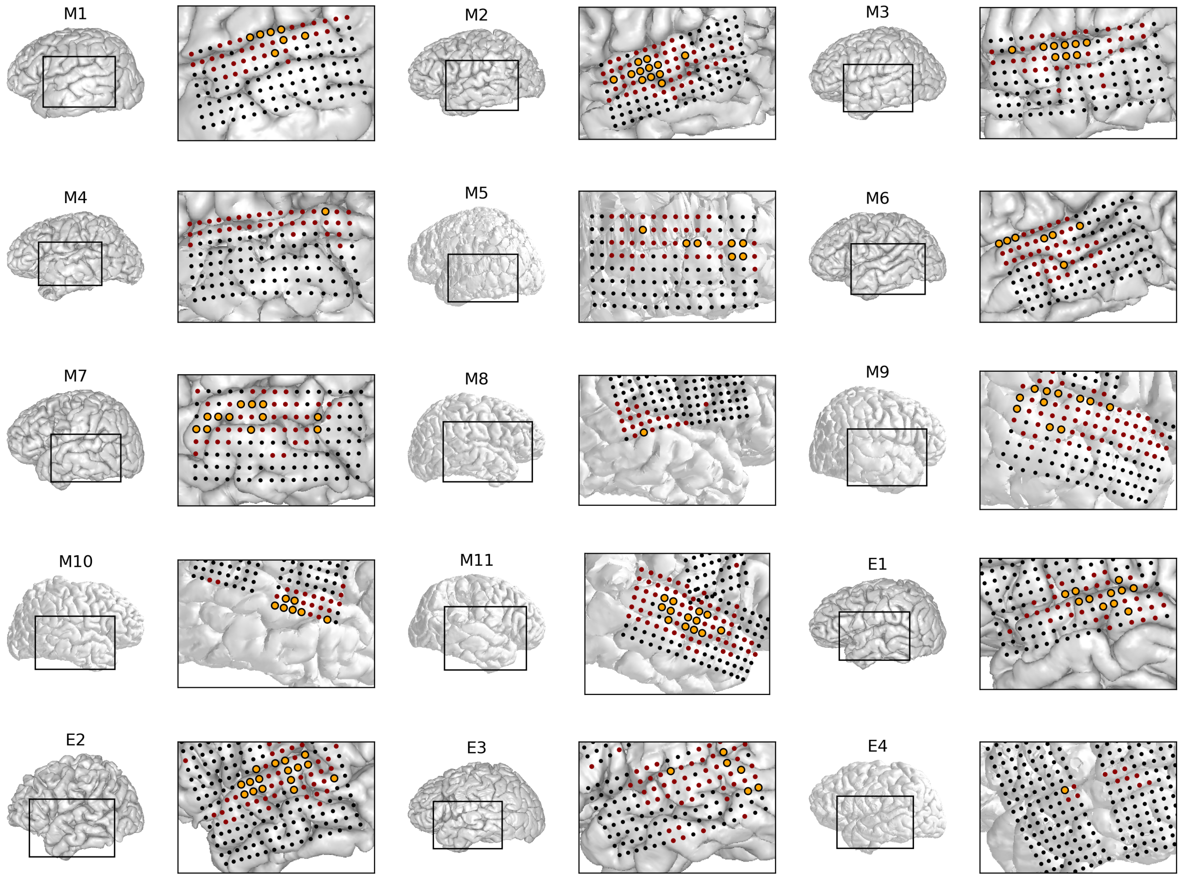

Supplementary Figure 3. Speech responsive and tone discriminating electrodes for all participants (native Mandarin speakers: M1-M11; native English speakers: E1-E4). ECoG grids covered the lateral temporal lobe of all participants. Dark red indicates electrodes that are responsive to speech. Circled orange electrodes indicate tone discriminating electrodes.

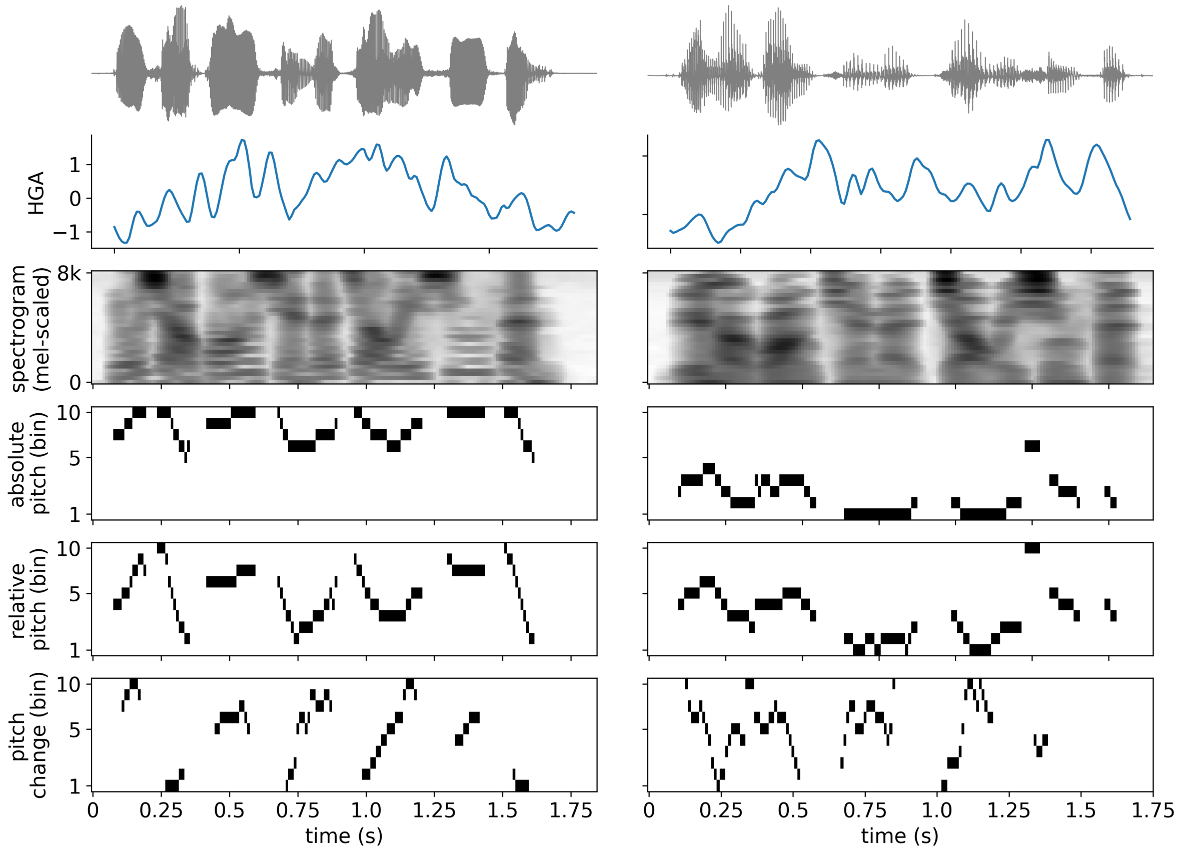

Supplementary Figure 4. Examples of feature extraction. Left and right columns represent the same sentence spoken by two different speakers (left column is a female speaker, right column is male) and different set of features all aligned to the same time. First row: raw waveform; second row: high-gamma (z-scored) activity at an example electrode; third row: Mel-scaled spectrogram; fourth row: absolute pitch (binned into 10 bins); fifth row: relative pitch (binned into 10 bins); last row: pitch change (binned into 10 bins).

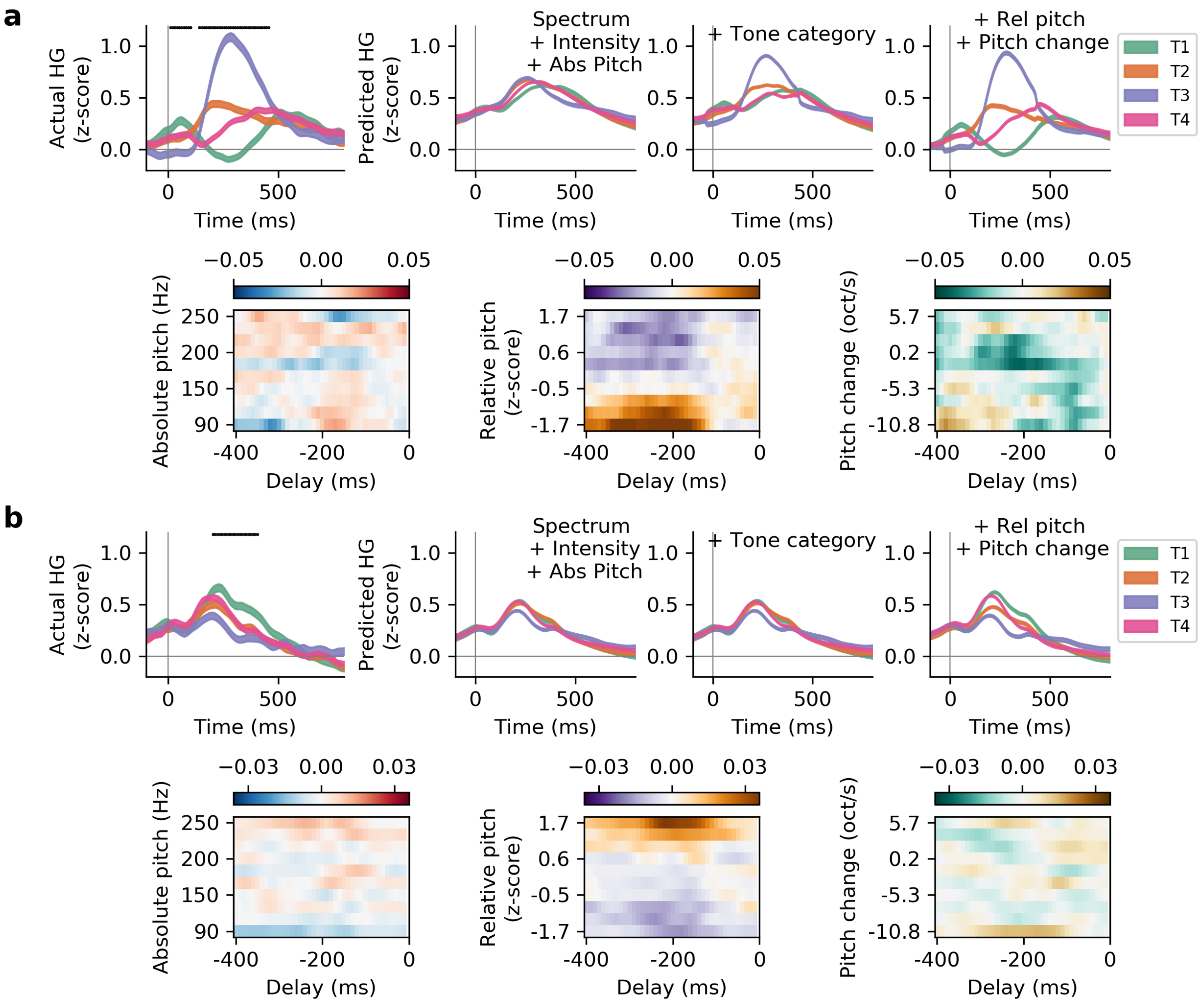

Supplementary Figure 5. Examples of different types of speaker-normalized pitch feature encoding. (a) Top row shows actual high-gamma responses and predicted responses from a single example electrode that differentiates lexical tones. The second to fourth panels of the top row show predicted responses from encoding models where tone-category and speaker-normalized pitch features are both left out (second panel), including tone category features (third panel), and including both tone category and speaker normalized pitch features (fourth panel), respectively. Bottom row shows regression weights from the model, indicating increased activity to low relative pitch height. **(b)** Same as **a**, but for an electrode tuned to high relative pitch.

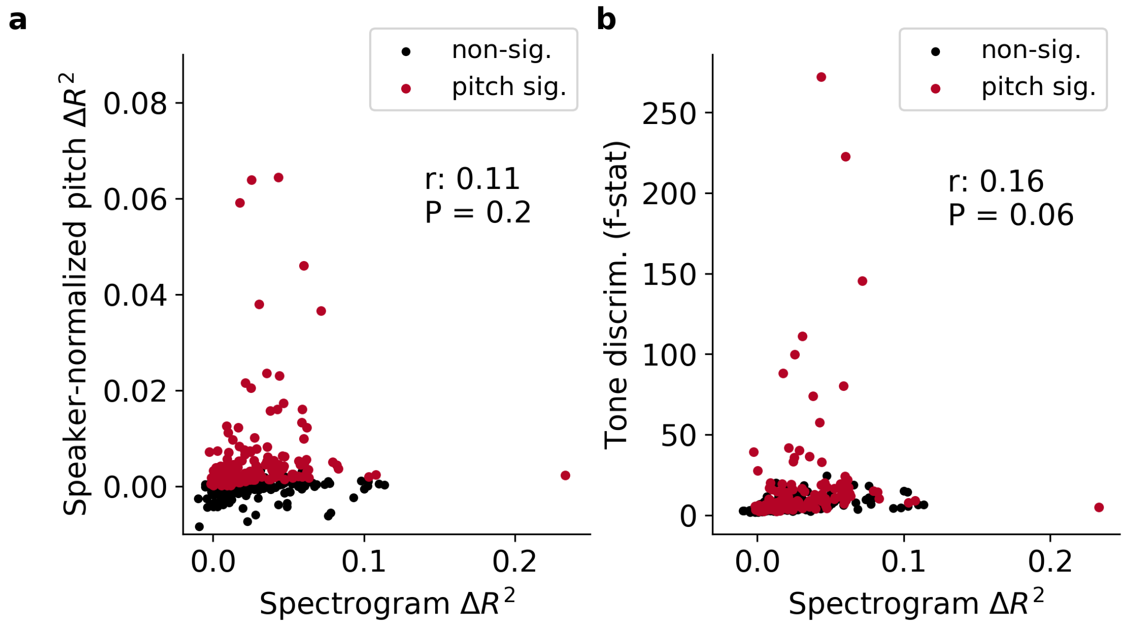

Supplementary Figure 6. Encoding of speaker-normalized relative pitch height and pitch change versus spectrogram features. **(a)**: Scatterplot of the unique variance explained by spectrogram features and by speaker-normalized pitch features, across speech-responsive electrodes from all Mandarin-speaking participants (M1-M11). Each dot represents a single electrode. Colored dots indicate significant encoding of speaker-normalized pitch (red; $p < 0.01$, permutation test). No significant correlation was found between spectrogram encoding and speaker-normalized pitch encoding. **(b):** Unique variance explained by speaker-normalized pitch features and tone discriminability. Red dots indicate electrodes that had significant encoding of speaker-normalized pitch features ($p < 0.01$, permutation test). No significant correlation was found between spectrogram encoding and tone discrimination.

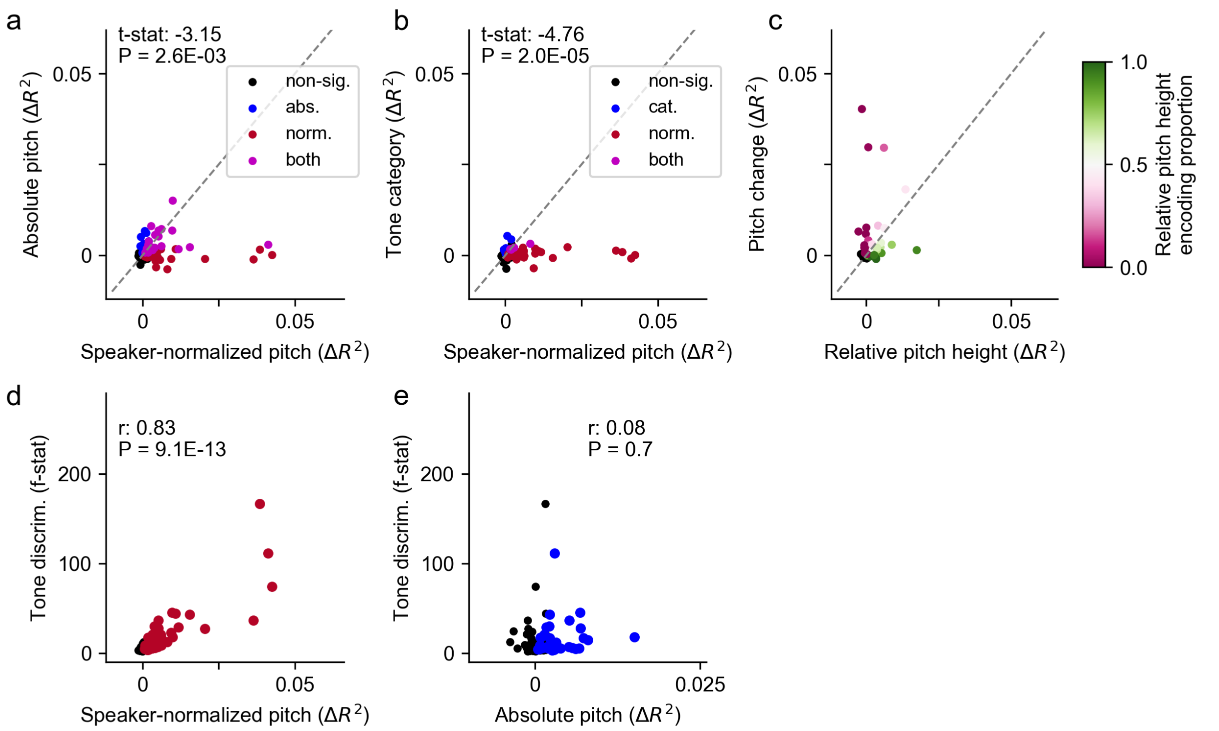

Supplementary Figure 7. Encoding of speaker-normalized relative pitch height and pitch change in left STG electrodes of native English speakers. (**a**) Scatterplot of the unique variance explained by absolute pitch and by speaker-normalized pitch features, across speech-responsive electrodes from all English-speaking participants (E1-E4). Each dot represents a single electrode. Colored dots indicate significant encoding of absolute pitch only (blue), speaker-normalized pitch only (red) or both (magenta). **(b)** Scatterplot comparing the relationship between the unique variance explained by speaker-normalized pitch features and discrete tone category features in the full encoding model in single electrodes. Colored dots indicate significant encoding of tone category only (blue), speaker-normalized pitch only (red) or both (magenta). **(c)** Scatterplot of the unique variance explained by relative pitch and by pitch change in single electrodes. Colored dots indicate significant encoding of either set of features, with the color indicating the proportion of the variance explained by the corresponding feature set. **(d)** Unique variance explained by speaker-normalized pitch features and tone discriminability. Colored dots indicate electrodes that had significant encoding of speaker-normalized pitch features ($p < 0.01$, permutation test). **(e)** Unique variance explained by absolute pitch and tone discriminability. Colored dots indicate electrodes that had significant encoding of absolute pitch ($p < 0.01$, permutation test). P-values in **(a, b, d, e)** are computed using two-sided t-test.

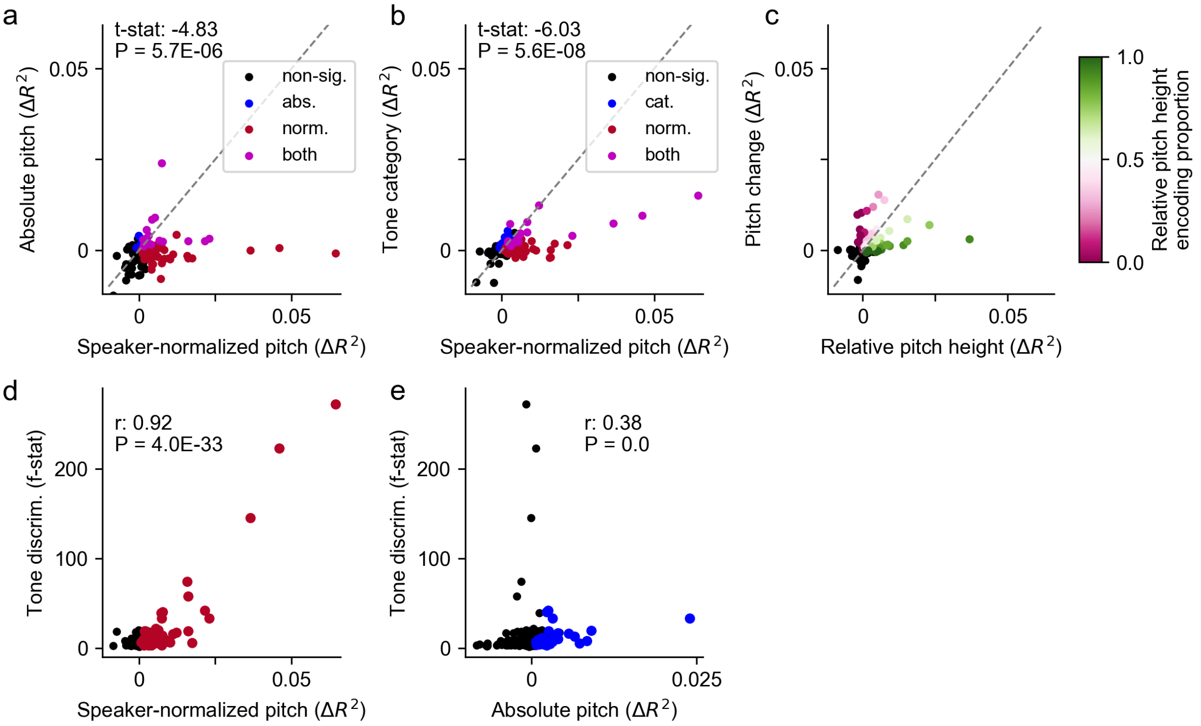

Supplementary Figure 8. Encoding of speaker-normalized relative pitch height and pitch change in left STG electrodes of native Mandarin speakers. (**a**) Scatterplot of the unique variance explained by absolute pitch and by speaker-normalized pitch features, across speech-responsive electrodes from Mandarin-speaking participants with left hemisphere coverage (M1-M7). Each dot represents a single electrode. Colored dots indicate significant encoding of absolute pitch only (blue), speaker-normalized pitch only (red) or both (magenta). **(b)** Scatterplot comparing the relationship between the unique variance explained by speaker-normalized pitch features and discrete tone category features in the full encoding model in single electrodes. Colored dots indicate significant encoding of tone category only (blue), speaker-normalized pitch only (red) or both (magenta). **(c)** Scatterplot of the unique variance explained by relative pitch and by pitch change in single electrodes. Colored dots indicate significant encoding of either set of features, with the color indicating the proportion of the variance explained by the corresponding feature set. **(d)** Unique variance explained by speaker-normalized pitch features and tone discriminability. Colored dots indicate electrodes that had significant encoding of speaker-normalized pitch features ($p < 0.01$, permutation test). **(e)** Unique variance explained by absolute pitch and tone discriminability. Colored dots indicate electrodes that had significant encoding of absolute pitch ($p <$ 0.01, permutation test). P-values in **(a, b, d, e)** are computed using two-sided t-test.

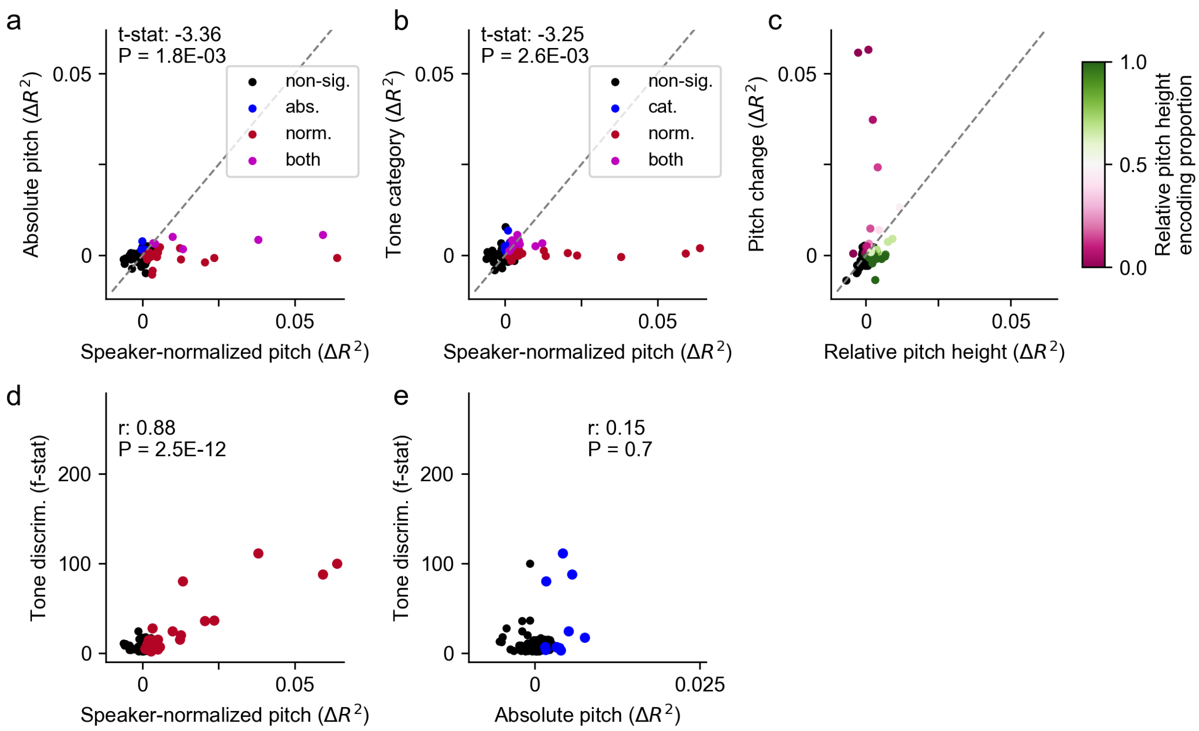

Supplementary Figure 9. Encoding of speaker-normalized relative pitch height and pitch change in right STG electrodes of native Mandarin speakers. (**a**) Scatterplot of the unique variance explained by absolute pitch and by speaker-normalized pitch features, across speech-responsive electrodes from all Mandarin-speaking participants with right hemisphere coverage (M8-M11). Each dot represents a single electrode. Colored dots indicate significant encoding of absolute pitch only (blue), speaker-normalized pitch only (red) or both (magenta). **(b)** Scatterplot comparing the relationship between the unique variance explained by speaker-normalized pitch features and discrete tone category features in the full encoding model in single electrodes. Colored dots indicate significant encoding of tone category only (blue), speaker-normalized pitch only (red) or both (magenta). **(c)** Scatterplot of the unique variance explained by relative pitch and by pitch change in single electrodes. Colored dots indicate significant encoding of either set of features, with the color indicating the proportion of the variance explained by the corresponding feature set. **(d)** Unique variance explained by speaker-normalized pitch features and tone discriminability. Colored dots indicate electrodes that had significant encoding of speaker-normalized pitch features ($p < 0.01$, permutation test). **(e)** Unique variance explained by absolute pitch and tone discriminability. Colored dots indicate electrodes that had significant encoding of absolute pitch ($p <$ 0.01, permutation test). P-values in **(a, b, d, e)** are computed using two-sided t-test.

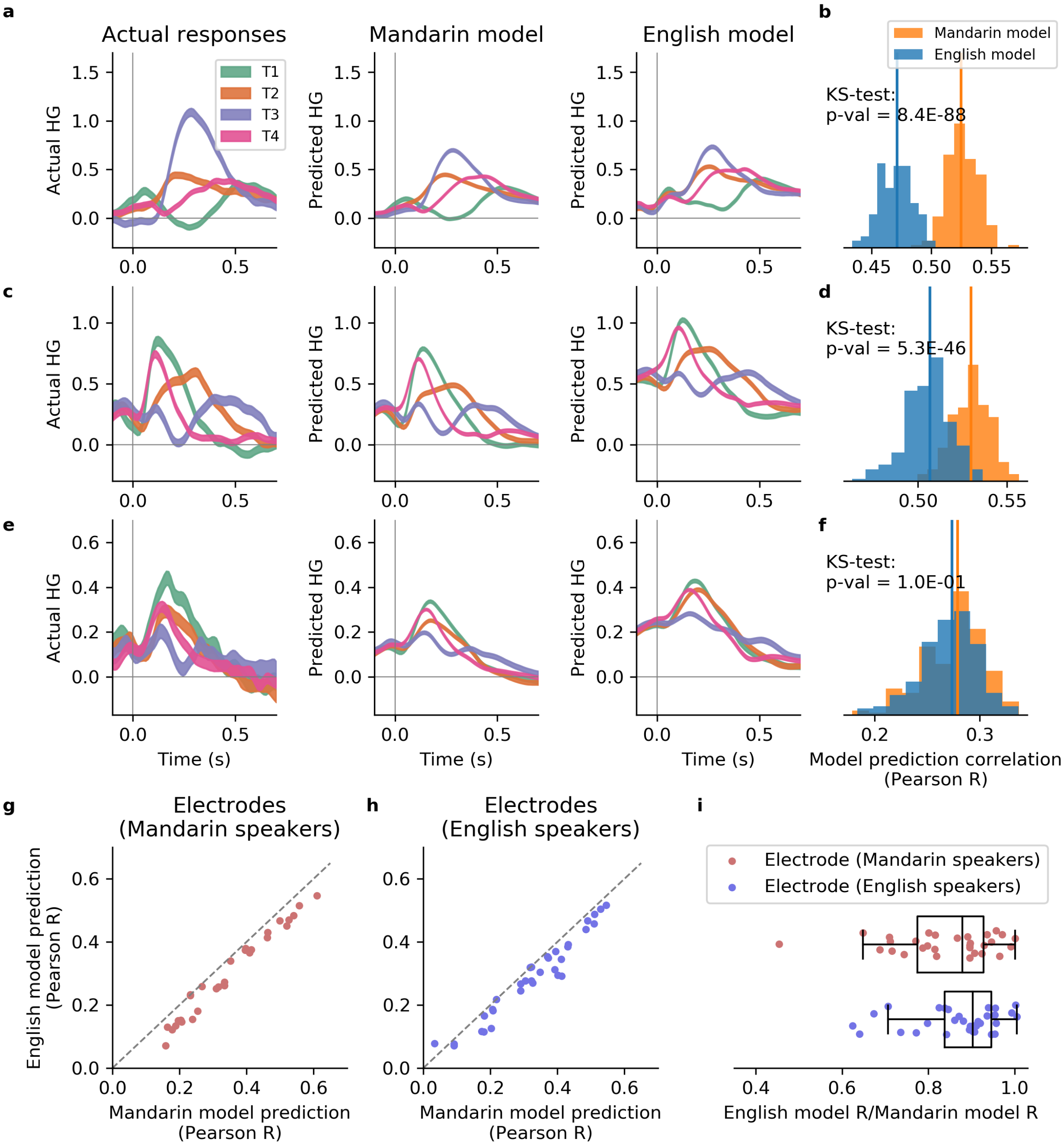

Supplementary Figure 10. Comparisons between models fit on Mandarin data and models fit on English data for both Mandarin and English speakers using Mandarin corpus. (a) Predicted responses and actual responses for an example electrode from a Mandarin-speaking participant. The left panel shows predicted responses from a model fit on Mandarin data while the right panel shows predicted responses from a model fit on English data. **(b)** Distribution of bootstrapped correlations between actual and predicted responses, n = 200 bootstrapped samples for each distribution. The correlations from the Mandarin model are significantly better than the correlations from the English model in this example, two-tailed Kolmogorov–Smirnov test (K-S test). **(c, d)** Same as **a, b**, but for an example electrode from an

English-speaking participant who does not speak Mandarin. Two-tailed K-S test. **(e, f)** Same panels as above, but showing an electrode from a Mandarin-speaking participant where the English model performs as well as the Mandarin model, two-tailed K-S test. **(g)** Scatter plot showing the correlation between actual and predicted responses for the Mandarin and English models for all tone-selective electrodes from Mandarin-speaking participants. **(h)** Same as **g**, but for tone-selective electrodes from English-speaking participants. **(i)** The fraction of English model correlation over Mandarin model correlation. The box plot shows median, 1st and 3rd quantiles, and the whiskers indicate 1.5 times of the interquartile range beyond the 1st and 3rd quantiles. $N = 33$ and 30 respectively.

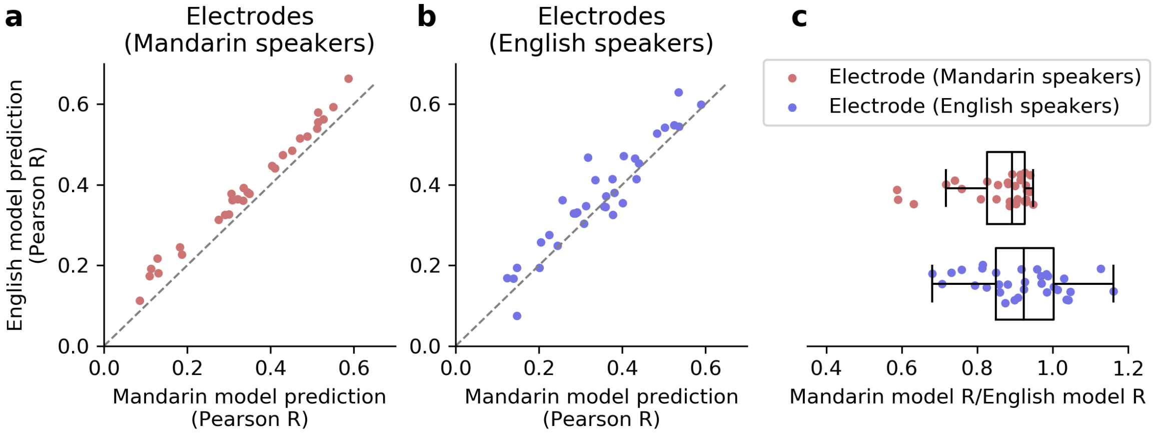

Supplementary Figure 11. Comparisons between models fit on Mandarin data and models fit on English data for both Mandarin and English speakers using English corpus. (a) Scatter plot showing the correlation between actual and predicted responses to English corpus for the Mandarin and English models for all tone-selective electrodes from Mandarin-speaking subjects. **(b)** Same as **a**, but for tone-selective electrodes from English-speaking participants. **(c)** The fraction of Mandarin model correlation over English model correlation. The box plot shows median, 1st and 3rd quantiles, and the whiskers indicate 1.5 times of the interquartile range beyond the 1st and 3rd quantiles.

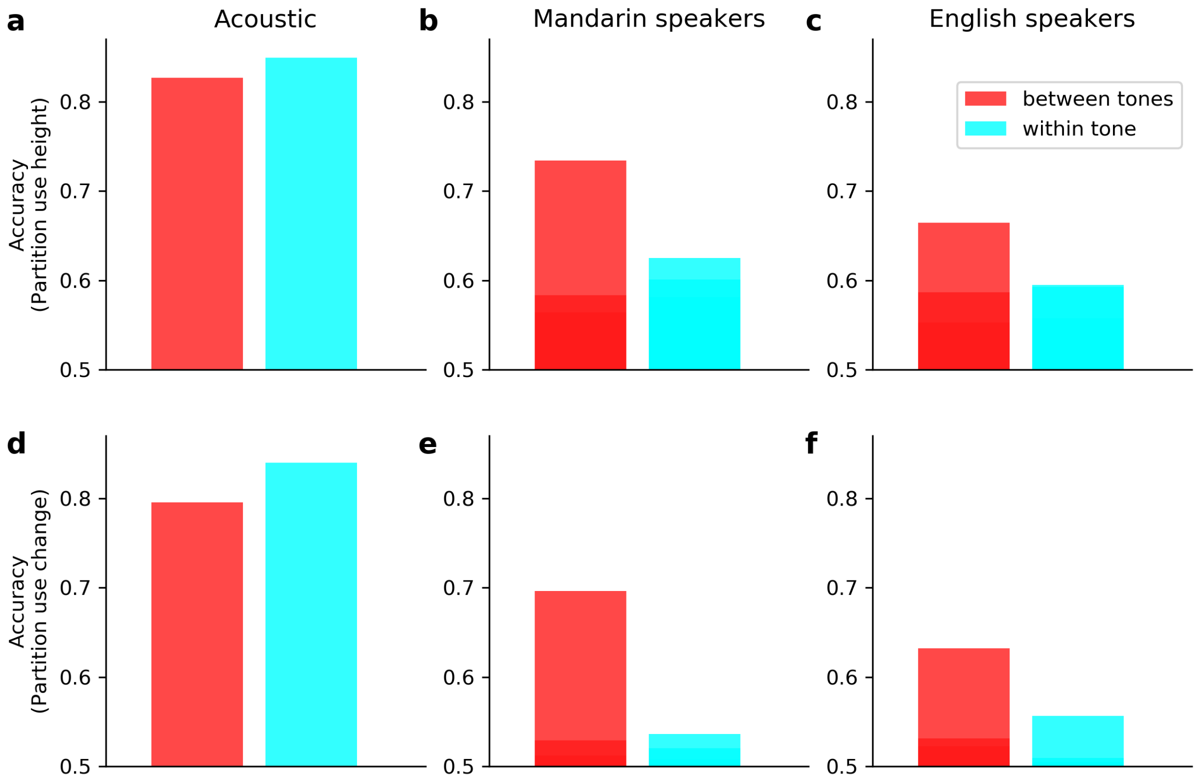

Supplementary Figure 12. Mean pairwise classification accuracy of between-tone groups (red), within-tone groups (cyan) in acoustic space (a, d), speech-responsive STG population space at peak time in native Mandarin speakers (b, e) and native English speakers (c, f) respectively. Top row **(a-c)** corresponds to results using relative pitch height partition; Bottom row **(d-f)** corresponds to results using pitch change partition (all accuracy in this figure has $p < 0.005$, permutation test, $p = 0.005$ corresponds to 0.521).

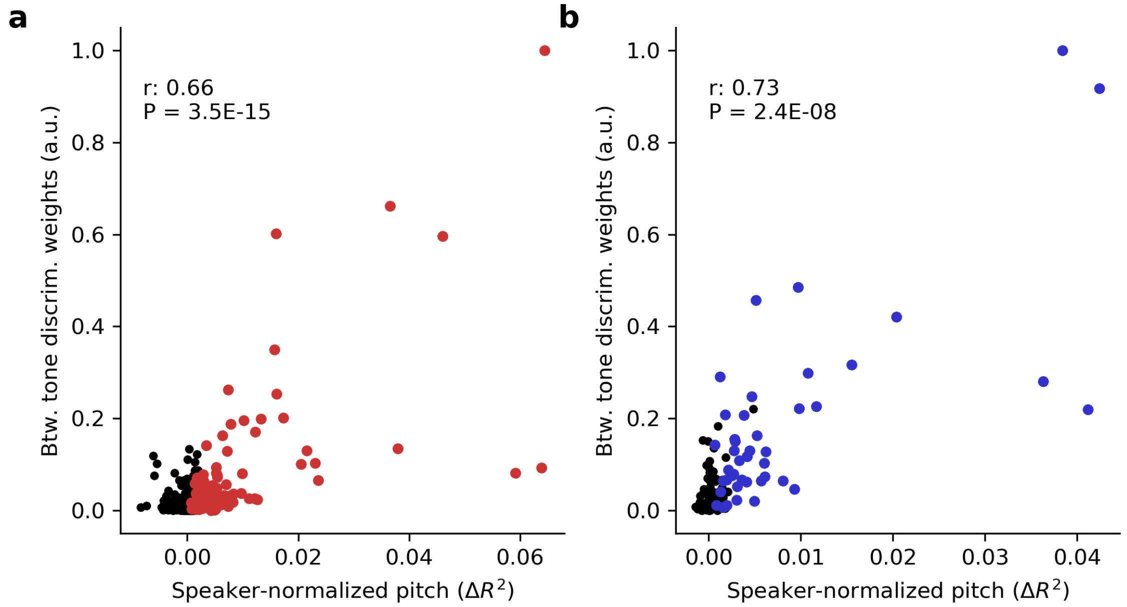

Supplementary Figure 13. Relationship between tone decoding and feature encoding at individual electrodes. (**a**) Scatterplot of the unique variance explained by speaker-normalized pitch and the averaged absolute weights in the between tone classifiers, across speech-responsive electrodes from all Mandarin-speaking participants. Each dot represents a single electrode. Colored dots indicate significant encoding electrodes. P-value is calculated using two-sided t-test. (**b**) The same as **a**, for speech-responsive electrodes from all English-speaking participants. P-value is calculated using two-sided t-test.

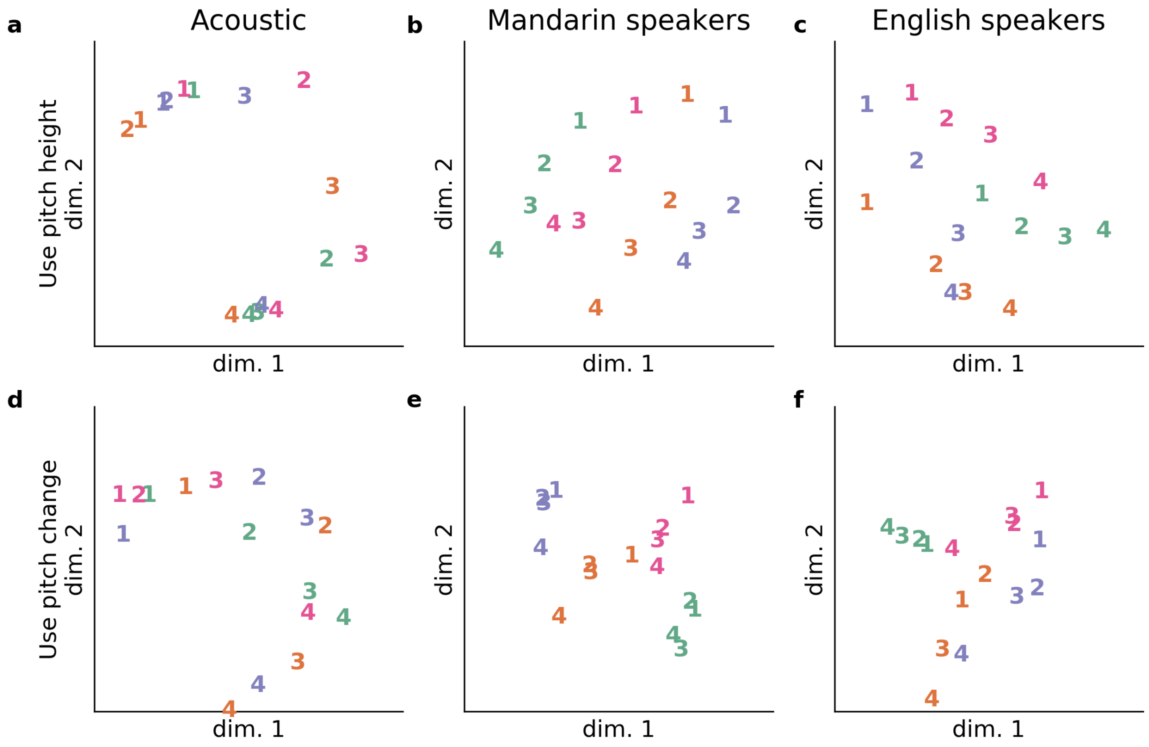

Supplementary Figure 14. **The relational organization of relative pitch contour and neural activity patterns using multi-dimensional scaling (MDS).** Based on the pairwise classification between the 16 groups of lexical tones (see Fig. **5g** and Methods for how tone exemplars were partitioned) in the acoustic pitch space (the contour of relative pitch height and pitch change) and in the neural space (the population response in speech-responsive electrodes), the representation dissimilarity matrices (RDMs) of the 16 groups were constructed in both the acoustic and the neural space. Using MDS, we transformed the RDMs into 2D visualizations that kept the pairwise dissimilarity between groups.
(a-c) include results based on the relative pitch height partition, and **(d-f)** include results based on pitch change partition. The three columns correspond to results in the acoustic pitch space **(a, d)**, the neural space in Mandarin speakers **(b, e)**, and neural space in English speakers **(c, f)**, respectively. In each panel, each colored number represents a partition group, with the colors indicating lexical tone identity (T1-green, T2-orange, T3-purple, T4-pink) and the value indicating the index of the group in the partition (see Fig. **5g**). The distance between a pair of numbers is proportional to the corresponding pairwise classification accuracy between groups. Overlapping numbers indicate confusion between groups (i.e., chance-level classification accuracy).

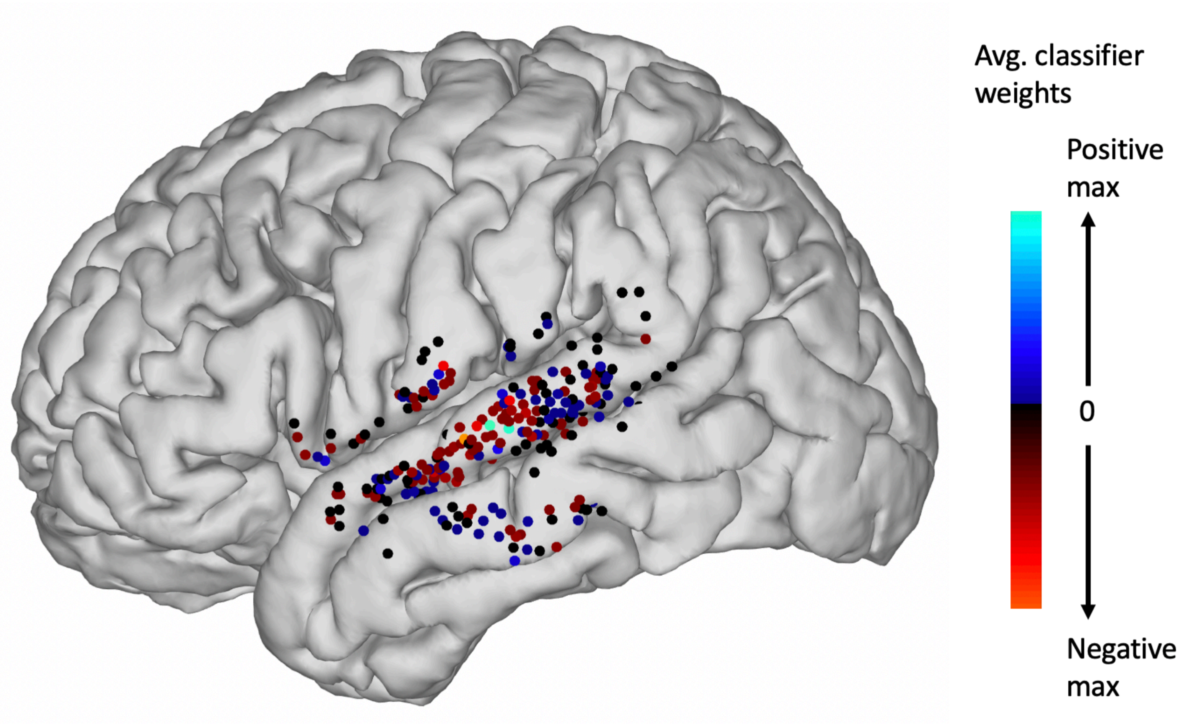

Supplementary Figure 15. Visualizing the averaged between tone classification weights in a warped common space for Mandarin speakers. The averaged between-tone classification weights (averaged across all 6 pair-wise classifiers) at peak time of classification accuracy were plotted on a common brain for all speech-responsive electrodes. Blue color indicates positive weights and red color indicates negative weights. Darker color indicates smaller absolute value and black corresponds to 0.

Supplementary Tables

Supplementary Table 1. A summary of number of task blocks finished by each subject.

Subject	Left/Right coverage	ASCCD (6 total blocks)	TIMIT (5 total blocks)	BURSC (6 total blocks)
E1	L	6	5	6
E2	L	6	5	6
E3	L	6	5	6
E4	L	5	5	
M1	L	6		
M2	L	6	2	1
M3	L	6	2	1
M4	L	6	2	1
M5	L	6	2	1
M6	L	6		
M7	L	6		
M8	R	4		
M9	R	4		
M10	R	4		
M11	R	4		
